# Acrylamide inhibits vaccinia virus through vimentin‐independent anti‐viral granule formation

**DOI:** 10.1111/cmi.13334

**Published:** 2021-05-03

**Authors:** Jennifer J. Wood, Ian J. White, Jerzy Samolej, Jason Mercer

**Affiliations:** ^1^ MRC Laboratory for Molecular Cell Biology, University College London London UK; ^2^ Institute of Microbiology and Infection, University of Birmingham Birmingham UK

**Keywords:** acrylamide, AVGs, poxvirus, vaccinia, VACV, vimentin

## Abstract

The replication and assembly of vaccinia virus (VACV), the prototypic poxvirus, occurs exclusively in the cytoplasm of host cells. While the role of cellular cytoskeletal components in these processes remains poorly understood, vimentin—a type III intermediate filament—has been shown to associate with viral replication sites and to be incorporated into mature VACV virions. Here, we employed chemical and genetic approaches to further investigate the role of vimentin during the VACV lifecycle. The collapse of vimentin filaments, using acrylamide, was found to inhibit VACV infection at the level of genome replication, intermediate‐ and late‐gene expression. However, we found that CRISPR‐mediated knockout of vimentin did not impact VACV replication. Combining these tools, we demonstrate that acrylamide treatment results in the formation of anti‐viral granules (AVGs) known to mediate translational inhibition of many viruses. We conclude that vimentin is dispensable for poxvirus replication and assembly and that acrylamide, as a potent inducer of AVGs during VACV infection, serves to bolster cell's anti‐viral response to poxvirus infection.

## 1 INTRODUCTION

Poxviruses are a family of large, enveloped dsDNA viruses that replicate exclusively in the cytoplasm of host cells (Moss 2013). Like all poxviruses the prototype member of this family, vaccinia virus (VACV), houses its viral genome and early transcription system in a dumbbell‐shaped core flanked by two proteinaceous structures termed lateral bodies (LBs) (Moss, 2013). The core and LBs are further encompassed by a single lipid bilayer viral membrane. VACV enters host cells by triggering macropinocytosis followed by acid‐mediated fusion from late compartments (Mercer & Helenius, 2008). Upon fusion, viral cores and LBs are delivered into the host cell cytoplasm (Schmidt et al., 2013). Early gene transcription is initiated within viral cores and LB proteins—thought to play an effector function—are dispersed (Schmidt et al., 2013). Following early gene expression, viral genomes are uncoated (Kilcher et al., 2014) and DNA replication initiated at multiple locations forming ER‐bound replication compartments—where genome replication, intermediate and late viral gene transcription and translation occur (Lin & Evans, 2010; Tolonen, Doglio, Schleich, & Locker, 2001). As replication compartments grow and merge, the ER is lost and new virus particles are assembled before leaving replication sites (Condit, Moussatche, & Traktman, 2006).

Being amongst the most complex mammalian viruses, poxviruses engage with a large repertoire of host proteins and processes to ensure successful infection and spread (Mercer et al., 2012). As such, VACV has become a useful to tool for investigation of basic virus‐host interactions and for the development of anti‐poxviral strategies (Bidgood, 2019). It is well accepted that VACV modulates the two main components of the host cytoskeleton (actin and microtubules) throughout its lifecycle. Dramatic reorganisation of host cell actin occurs during virus entry (Mercer et al., 2010; Mercer & Helenius, 2008) and egress (Cudmore, Cossart, Griffiths, & Way, 1995; Smith & Law, 2004), and microtubules are subjugated to coalesce transcription and replication sites (Mallardo, Schleich, & Locker, 2001), to transport nascent virions from assembly to wrapping compartments and subsequently to the cell surface (Rietdorf et al., 2001; Ward & Moss, 2001).

It has also been reported that VACV infection results in reorganisation of vimentin, a type III intermediate filament involved in a range of cellular functions including; cell migration, proliferation, signal transduction and the organisation of cytosolic organelles (Chang & Goldman, 2004; Danielsson, Peterson, Araujo, Lautenschlager, & Gad, 2018; Ivaska, Pallari, Nevo, & Eriksson, 2007; Lowery, Kuczmarski, Herrmann, & Goldman, 2015; Minin & Moldaver, 2008; Styers et al., 2004). For VACV, vimentin filaments were shown to surround and concentrate within viral replication sites (Risco et al., 2002), leading to the hypothesis that it plays a role in their formation and in the assembly of early virion intermediates (Risco et al., 2002). In support of this hypothesis, vimentin was identified in proteomics analyses of purified mature VACV virions, suggesting that this intermediate filament was associated with, or packaged into, viral particles during assembly (Chung et al., 2006; Resch, Hixson, Moore, Lipton, & Moss, 2007).

In the absence of small compounds that specifically depolymerise vimentin, acrylamide—which disrupts vimentin polymerisation without impacting other cytoskeletal elements (Durham, Pena, & Carpenter, 1983; Eckert, 1986)—is often employed to study the interplay between viruses and vimentin. In addition to its impact on vimentin, acrylamide treatment has been shown to activate several oxidative and ER stress pathways (Komoike & Matsuoka, 2016). Exposure to acrylamide results in the production of reactive oxygen species and mis‐regulation of cellular redox status (Jiang et al., 2007; Kim, Park, Rhee, & Pyo, 2015), which ultimately triggers eIF2α signalling and a shift from global to stress specific protein synthesis (Jackson, Hellen, & Pestova, 2010; Komoike & Matsuoka, 2016; Komoike & Matsuoka, 2019). Using acrylamide as a tool, vimentin has been reported to play diverse roles in the lifecycles of several viruses including: Chikungunya virus (Issac, Tan, & Chu, 2014), HIV (Wang, Zhang, Han, Wang, & Gao, 2016), HCMV (Miller & Hertel, 2009), MVM Parvovirus (Fay & Pante, 2013), Junin (Cordo & Candurra, 2003) and bluetongue virus (Bhattacharya, Noad, & Roy, 2007).

Here, we set out to investigate the role of vimentin in VACV replication and assembly. In line with previous reports (Resch et al., 2007; Risco et al., 2002), we observed that during VACV infection vimentin associates with replication sites and is packaged into newly assembled virions. We demonstrate that treatment of infected cells with acrylamide, which correlates with the collapse of vimentin filaments, dramatically impacts VACV production through inhibition of genome replication and subsequent gene expression. Surprisingly, upon generating a vimentin‐null cell line, we found that infection was still sensitive to acrylamide and that viral replication was not altered by the loss of vimentin. Consistent with a vimentin‐independent effect, we show that acrylamide treatment leads to the formation of anti‐viral granules (AVGs), which appear to block post‐replicative gene expression and the production of new virus particles.

## 2 RESULTS

### 2.1 Vimentin is peripheral to VACV replication sites and within mature virions

**FIGURE 1:**
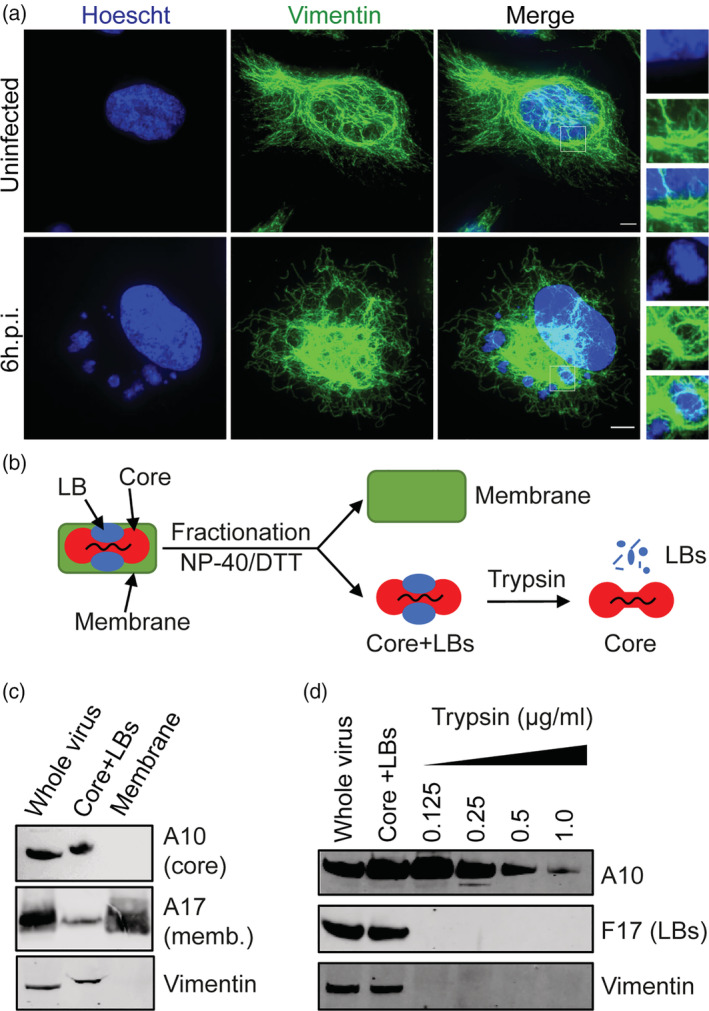
Vimentin is associated with VACV replication. (a) HeLa cells were infected with WR VACV at MOI 10 for 6 hr then fixed and stained with anti‐vimentin (green). DNA‐visualised using Hoechst. Scale bars = 5 μm. (b) Illustration of VACV fractionation protocol. Intact purified virions were subjected to treatment with detergent and reducing agents to separate viral membranes and core+LBs. To remove LBs, Core+LBs fractions were treated with trypsin (details in M and Ms). (c) Representative western blot of purified WT WR VACV virions, VACV core+LBs and membrane fractions as prepared per (b). Fractions were immunoblotted for A10 (core protein), A17 (membrane protein) and vimentin. (d) Representative western blot of purified WT WR VACV virions fractionated, and LBs removed as per (b) using increasing concentrations of trypsin. Samples were immunoblotted for A10 (core), F17 (LB) and vimentin. LBs, lateral bodies; VACV, vaccinia virus

The intermediate filament vimentin has been observed to be associated with VACV replication sites by electron and fluorescence microscopy (Resch et al., 2007, Risco et al., 2002). To verify the rearrangement and relocalisation of vimentin to sites of VACV replication, HeLa cells were infected with VACV and at 6 hr post infection (hpi) cells were stained with Hoechst to visualise viral replication sites and immunolabelled for vimentin. In uninfected control cells, vimentin appeared as a tubular network localised around the nucleus of the cell (Figure 1a; top). In VACV infected cells, as previously reported, we found vimentin in close association with the periphery of multiple viral replication sites present in the cytoplasm (Figure 1a; bottom see ROI).

Next, we sought to confirm the presence of vimentin within purified virions. While vimentin has been observed within assembling cytoplasmic virions by immuno‐electron microscopy (EM) (Risco et al., 2002), its presence within mature virions (MVs) as determined by mass spectrometry is debated (Chung et al., 2006; Resch et al., 2007). To confirm and extend these findings, purified wild‐type VACV MVs were subjected to fractionation into core+LB and membrane samples as previously described (Mercer & Traktman, 2003). Whole virions, core‐LB and membrane fractions were then subjected to immunoblot analysis directed against the core protein A10, the membrane protein A17 and vimentin (Figure 1b). Separation of A10 into core+LB and A17 into membrane samples indicated that fractionation was successful. Vimentin was present in whole virus and, upon fractionation, exclusively found in the core+LB sample (Figure 1c; bottom panel). These results confirm the presence of vimentin in VACV virions and demonstrate its association with VACV cores or LBs.

To further refine its intra‐virion localisation, we subjected the core+LB fraction to increasing trypsin treatment to digest the LBs away from the viral cores; a procedure adapted from Ishihashi and Oie (Ichihashi, Oie, & Tsuruhara, 1984). As expected, at low trypsin concentrations the core protein A10 remained intact, while the LB protein F17 (Schmidt et al., 2013) was completely degraded (Figure 1d). Immunoblot analysis of vimentin mirrored that of F17, suggesting that vimentin is accessible and perhaps associated with LBs (Figure 1d; bottom panel).

### 2.2 Acrylamide inhibits VACV infection at intermediate and late transcription

**FIGURE 2:**
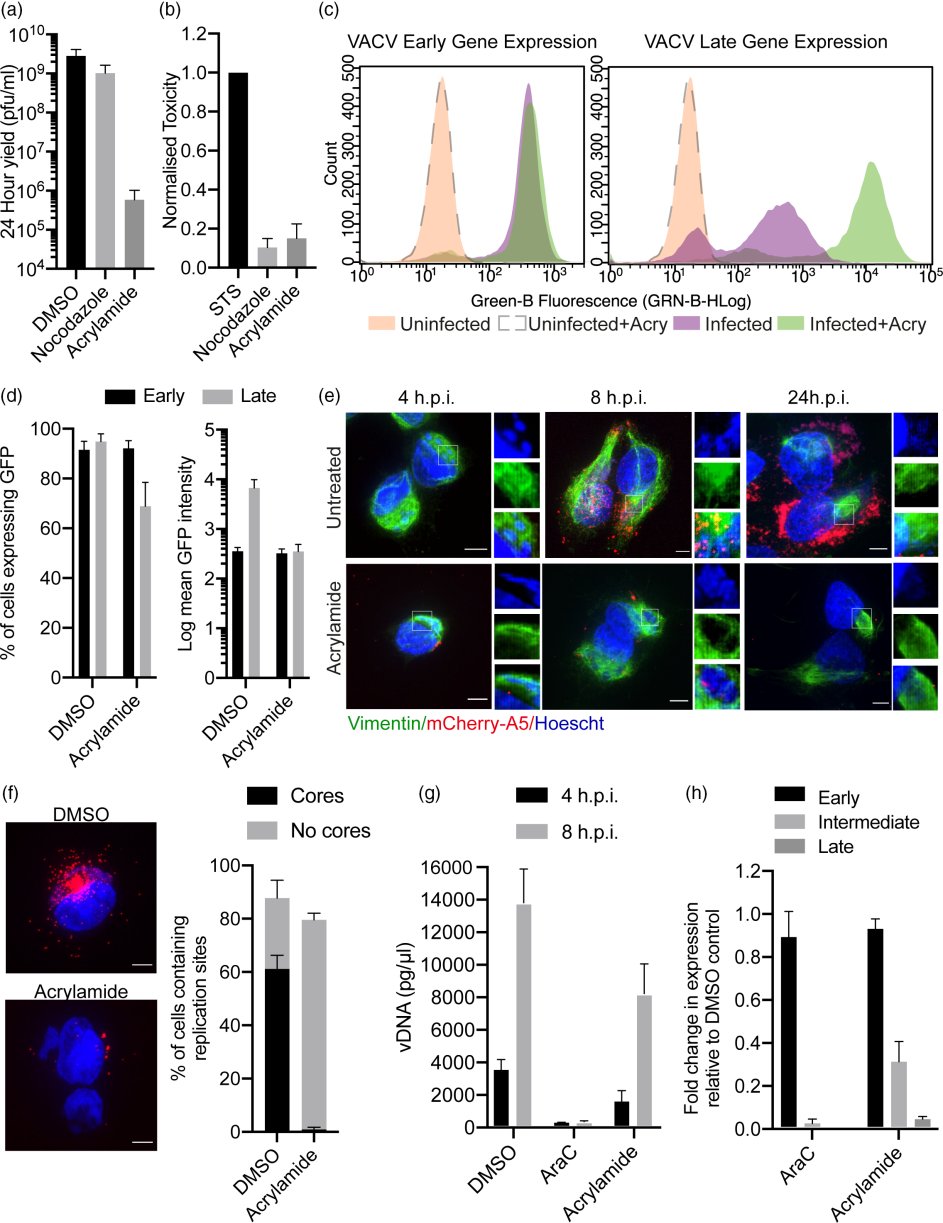
Acrylamide inhibits vaccinia virus (VACV) infection. (a) HeLa cells treated with either DMSO, nocodazole or acrylamide were infected with WT WR VACV at MOI 1 and viral yield determined at 24 hpi on BSC40 cells. (b) HeLa cells were incubated with staurosporine (STS), acrylamide or nocodazole for 24 hr and cytotoxicity measured using the Pierce LDH Cytotoxicity Assay Kit. Experiments were performed in triplicate and results normalised to STS. (c) HeLa cells were infected with WR E‐EGFP (left) or L‐EGFP VACV (right) at MOI 4, treated with DMSO or acrylamide and analysed by flow cytometry at 6 (early) or 8 (late) hpi. Representative flow traces of cell count vs. fluorescence intensity for uninfected (orange), uninfected with acrylamide (dashed), infected (green) and infected with acrylamide (purple) cell populations. (d) Mean percentage of EGFP expressing cells and Log EGFP intensity of three independent experiments from (c). (e) HeLa cells infected with WR mCh‐A5 (red) at MOI 10 in the presence of DMSO or acrylamide were fixed at 4, 8 and 24 hpi. Cells were immunostained for vimentin (green) and DNA was visualised using Hoechst (blue). Scale bars = 5 μm (f) HeLa cells were infected with WR mCh‐A5 (red) at MOI 10 in the presence of DMSO or acrylamide. At 8 hpi, cells were stained for DNA using Hoechst (blue). Scale bars = 5 μm. The percentage of cells containing replication sites with and without new virions was quantified (*n* > 75). (g) To quantify VACV genome replication, HeLa cells were infected with WT WR at MOI 10 in the presence of DMSO, AraC or acrylamide. Genomic DNA (at 4 and 8 hpi) was extracted and quantified by qPCR. (h) HeLa cells were infected as in (G) and samples harvested at 2, 4 or 6 hpi. To quantify vRNA levels RTqPCR was performed using early, intermediate or late gene specific primers. GAPDH was used to normalise expression across all samples and fold change in vRNA calculated using threshold cycles. All experiments were performed in triplicate with graphs representing the mean + 1 SD

Based on its localisation during infection, Risco *et al*. proposed that vimentin plays a role in the formation for VACV replication sites and virions (Risco et al., 2002). Having confirmed these localisation studies, we wanted to test the importance of an intact vimentin network during VACV infection. With its ability to collapse vimentin filaments well documented (Durham et al., 1983; Miller & Hertel, 2009), we proceeded to evaluate the impact of acrylamide treatment on VACV infection. Cells were infected in the presence of acrylamide and the 24 hr viral yield determined. Strikingly, the viral yield in the presence of acrylamide was reduced by greater than three‐logs (Figure 2a). DMSO and Nocodazole, which destabilises microtubules, were used as controls. Treatment with either did not result in significant reduction in virus production consistent with previous reports (Ploubidou et al., 2000; Rizopoulos et al., 2015). To verify that this reduction in viral yield was not due to general toxicity of acrylamide, cytotoxicity assays were performed on HeLa cells incubated with acrylamide for 24 hr. Staurosporine (STS), a known inducer of apoptosis was used as a positive control and nocodazole, which did not impact virus yield, was used a negative control. While STS induced high levels of cytoxicity, acrylamide was comparable to nocodazole (Figure 2b) indicating that acrylamide‐associated toxicity was not responsible for the reduction in VACV production. Given the dramatic reduction in virus yield seen in the presence of acrylamide, we asked which stage of the VACV life cycle was inhibited.

VACV gene expression occurs in three temporal stages with early genes being expressed before genome replication within the virus core, and intermediate as well as late genes after genome replication (Moss, 2013). To determine if the acrylamide block was pre‐ or post‐ genome replication, we infected HeLa cells with recombinant viruses that express EGFP under the control of early (WR E‐EGFP) or late (WR L‐EGFP) viral promoters and analysed them for EGFP expression by flow cytometry (Yakimovich et al., 2017). When plotted as a histogram (cell number vs. fluorescence intensity), no defect in either the number of infected cells or intensity of EGFP expression was seen with WR E‐EGFP virus in the presence of acrylamide (Figure 2c; left panel). However, a dramatic reduction in the fluorescence intensity of cells infected with WR L‐EGFP was observed in the presence of acrylamide (Figure 2c; right panel). Quantification showed that neither the number or fluorescence intensity of WR E‐EGFP‐infected cells was affected by acrylamide, while the number of WR L‐EGFP‐infected cells was reduced by 26%, and the fluorescence intensity of these infected cells reduced by 1.3 logs (Figure 2d). That reduced levels of late gene expression were observed indicated that acrylamide blocks the VACV lifecycle after early gene expression.

To investigate post‐replicative stages of VACV infection, HeLa cells were infected with a fluorescent recombinant virus that expresses and packages a mCherry‐tagged version of the VACV core protein A5 (WR mCh‐A5) (Schmidt, Bleck, Helenius, & Mercer, 2011). Cells were left untreated or were treated with acrylamide and infection allowed to proceed for 4, 8 or 24 hr prior to fixation. Nuclei and viral replication sites were visualised using Hoechst, vimentin via immunostaining and virions through the incorporation of mCh‐A5. In untreated cells, at 4 hpi cytoplasmic replication sites surrounded by vimentin were seen (Figure 2e; top). By 8 hpi, nascent virions had formed, and the vimentin appeared more loosely associated with replication sites, a phenotype that was more pronounced at 24 hpi. Conversely, infected cells treated with acrylamide displayed a total collapse of vimentin filaments around viral replication sites at all time points (Figure 2e; bottom). Notably, in the presence of acrylamide, replication sites appeared qualitatively smaller than in control cells and no new virions were formed as indicated by a lack of robust mCherry core signal within replication sites. Quantification of the number of cells containing replication sites, and VACV cores within them, at 8 hpi showed that in the presence of acrylamide, replication sites could form, but no virions were assembled (Figure 2f).

Having observed smaller replication sites in the presence of acrylamide, we next quantified its effect on viral genome replication. HeLa cells were infected with WT VACV, in the presence or absence of acrylamide, and genomic DNA extracted at 4 and 8 hpi. The amount of VACV DNA was then quantified using qPCR as previously described (Huttunen & Mercer, 2019). Cytosine arabinoside (AraC), a known inhibitor of VACV DNA replication was included as a control (Balzarini & Declercq, 1989; Sidwell, Dixon, Sellers, & Schabel, 1968). As expected, there was a ~four‐fold increase in VACV DNA between 4 and 8 hpi in control cells, and only negligible amounts of vDNA in AraC‐treated samples (Figure 2g). We found that acrylamide did not completely inhibit VACV DNA accumulation but reduced the levels of VACV DNA, relative to DMSO controls, at both timepoints (Figure 2g). These results are consistent with our immunofluorescence images and suggest that acrylamide partially blocks or slows VACV DNA replication.

Having observed a ~two‐fold decrease in VACV DNA accumulation, but a 1.3 log decrease in late gene expression, we reasoned that the step in between the two may be the target of acrylamide‐mediated inhibition. To quantify the effect of acrylamide on VACV mRNA levels, we performed qRT‐PCR on canonical early (J2R), intermediate (G8R) and late (F17) genes (Huttunen & Mercer, 2019). Again, AraC‐treated infected cells were used to benchmark inhibition of intermediate and late gene transcription. In both AraC and acrylamide‐treated conditions, we observed no significant change in early gene transcription relative to the DMSO‐treated control, and as anticipated AraC abolished intermediate and late gene expression (Figure 2h). In the presence of acrylamide, we observed a three‐fold and >20‐fold drop in intermediate and late gene transcription, respectively (Figure 2h). These results indicated acrylamide inhibited post‐replication VACV gene transcription.

### 2.3 Vimentin is not required during VACV infection

**FIGURE 3:**
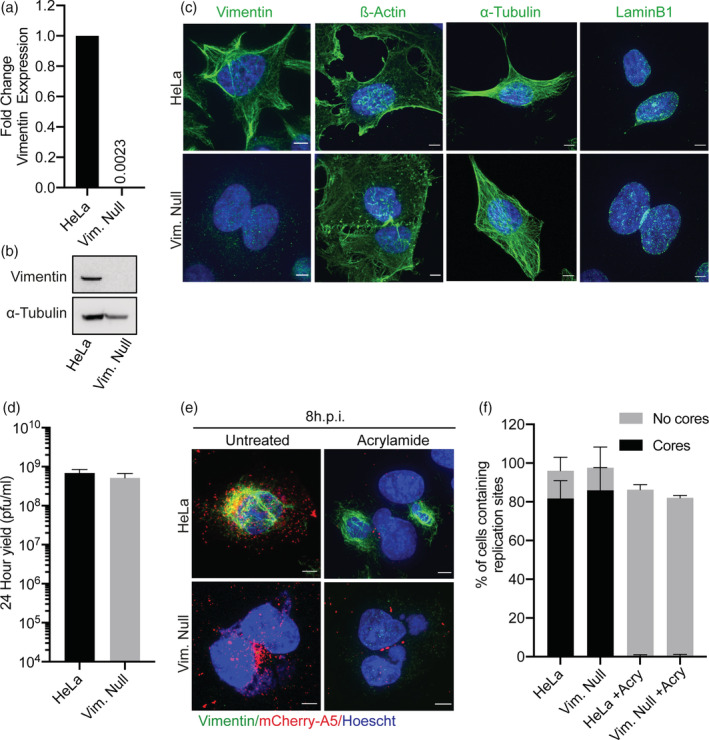
Acrylamide‐mediated inhibition of vaccinia virus (VACV) infection is not due to collapsing of vimentin filaments. (a) Quantification of vimentin mRNA levels in parental HeLa and HeLa Vimentin‐Null (Vim. Null) cells using RTqPCR. GAPDH was used to normalise expression across all samples and the fold change vimentin mRNA calculated using threshold cycles. (b) Representative immunoblot of HeLa and Vim. Null cell lysates immunoblotted for vimentin; α‐tubulin served as a loading control. (c) HeLa and Vim. Null cells were stained for cytoskeletal elements: α‐Tubulin, LaminB1 or β‐Actin and DNA‐visualised using Hoechst. Scale bars = 5 μm. (d) HeLa and Vim. Null cells were infected with WT WR at MOI 1 and viral yield determined at 24 hpi on BSC40 cells. (e) HeLa and Vim. Null cells were infected with WR mCh‐A5 (red) at MOI 10 in the absence or presence of acrylamide. At 8 hpi, cells were stained for vimentin (green), DNA (blue). Representative cell images shown. Scale bars = 5 μm. (f) The percentage of cells containing replication sites, with and without mCh‐A5 cores from (e) (*n* > 75). All experiments were performed in triplicate with graphs representing the mean + 1 SD

Risco *et al*. proposed that vimentin acts to coordinate replication site formation and virus assembly (Risco et al., 2002). As we saw a minor defect in replication site size and a primary defect in post‐replication gene transcription using acrylamide, we sought to determine if vimentin played an additional role in virus assembly. To this end, we generated a vimentin‐null HeLa cell line using CRISPR. The lack of vimentin expression was confirmed at the transcriptional level by qRT‐PCR (Figure 3a; top) and at the protein level by immunoblot (Figure 3b; bottom). Comparison of the actin, tubulin and lamin networks in parental and vimentin‐null cells, by immunofluorescence, revealed no major differences in network architecture, cell size or cell shape (Figure 3c).

Next, we sought to determine the ability of the vimentin‐null cells to support productive VACV infection using a 24 hr yield assay. To our surprise, we saw no difference in VACV infectious yield between parental and vimentin‐null cell lines (Figure 3d). This result indicated that vimentin is not required for productive VACV infection. In light of this observation, we speculated that acrylamide‐mediated inhibition of VACV might be due to the collapse of vimentin filaments constricting genome replication or impeding the spatial organisation of replication sites. To investigate this possibility, parental HeLa and vimentin‐null cells were infected with WR mCh‐A5 in the presence or absence of acrylamide. At 8 hpi, cells were fixed, nuclei and replication sites were stained with Hoechst and immunostaining was performed to visualise vimentin. In untreated HeLa cells, vimentin was associated with replication sites and newly assembled virions were found throughout the cytoplasm (Figure 3e). As expected, in acrylamide‐treated HeLa cells vimentin was collapsed around replication sites and no virions were formed. Consistent with the 24 hr yield results, both replication sites and newly formed virions were seen in vimentin‐null cells (Figure 3e). Contrary to our hypothesis however, treatment of vimentin‐null cells with acrylamide blocked the production of virions as effectively as in parental HeLa cells. Quantification of this phenotype indicated that regardless of the presence of vimentin, acrylamide treatment causes a small reduction in the number of cells with replication sites while completely abolishing the formation of nascent VACV virions (Figure 3f). Thus, using a vimentin‐null cell line, we demonstrate that inhibition of VACV infection by acrylamide is independent of its ability to collapse cell's vimentin network.

### 2.4 Acrylamide blocks translation and stimulates the formation of AVGs

In addition to collapsing vimentin, acrylamide has been reported to activate cellular oxidative and ER stress responses (Komoike & Matsuoka, 2016; Komoike & Matsuoka, 2019), which converge upon eIF2α phosphorylation and global translational arrest (Jackson et al., 2010; McCormick & Khaperskyy, 2017). Translational arrest, in turn leads to the formation of cytosolic stress granules (SGs) (Guzikowski, Chen, & Zid, 2019). Similar to SGs, AVGs, have been reported to form and restrict VACV gene expression. AVGs were reported to form in ~10% of WT infected cells, in cells infected with VACV strains that lack the ability to block PKR‐mediated responses and upon the addition of small compounds or anti‐virals that increase cellular stress (Rozelle, Filone, Kedersha, & Connor, 2014; Simpson‐Holley et al., 2011).

**FIGURE 4:**
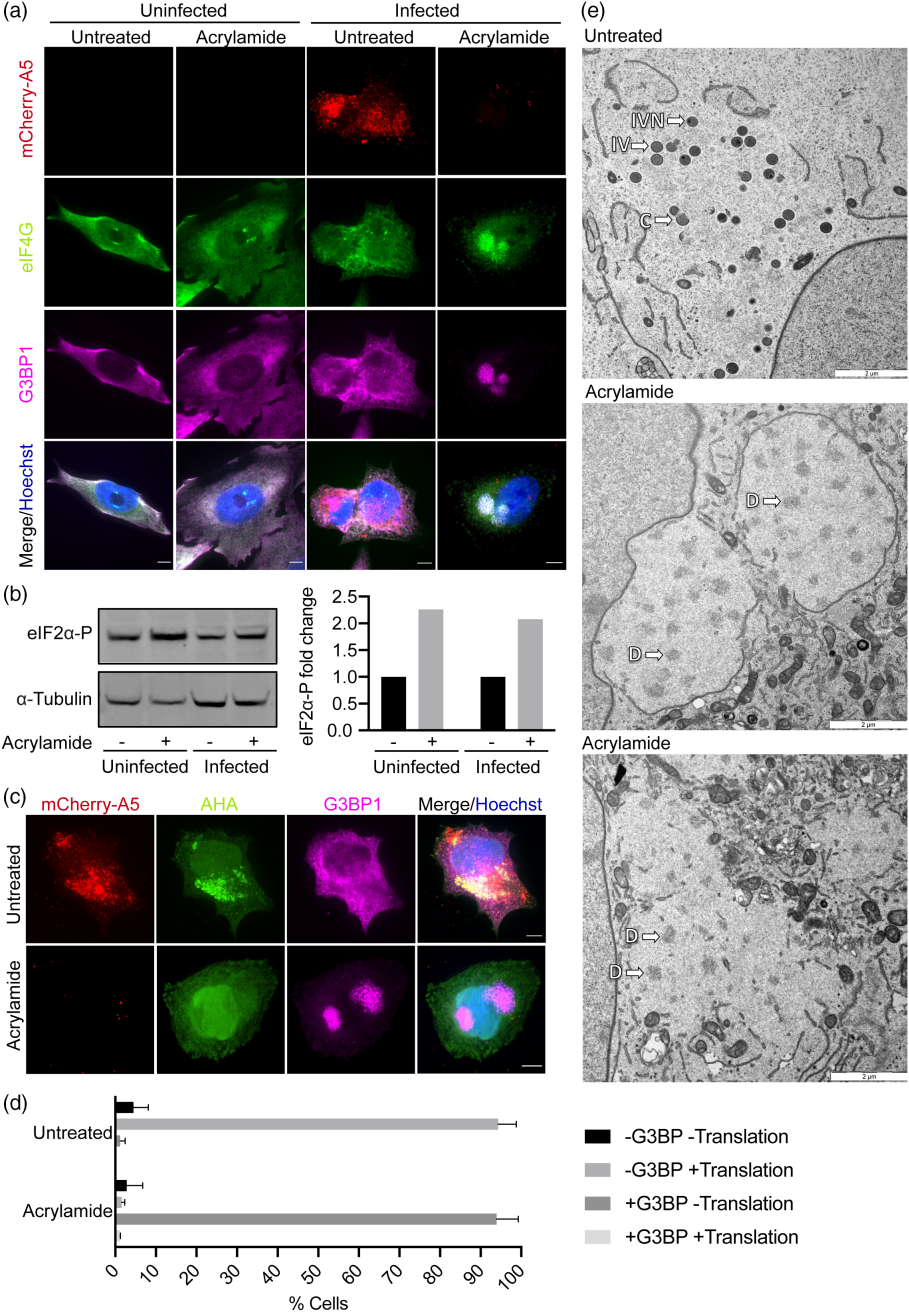
Acrylamide blocks translation and stimulates formation of anti‐viral granules. (a) HeLa cells were mock infected or infected with WR mCh‐A5 at MOI 10 in the absence (untreated) or presence of acrylamide. At 8 hpi, cells were fixed and stained for eIF4G and G3BP1, and DNA (blue). Scale bars = 5 μm. (b) HeLa cells were either mock infected or infected with WT WR at MOI 10 in the absence or presence of acrylamide. At 8 hpi, cells were harvested, lysed and immunoblotted for phospho‐eIF2α (left). Quantification of the fold‐change in phospho‐eIF2α observed upon acrylamide treatment is plotted (right). α‐tubulin served as a loading control and for quantification normalisation. (c) HeLa cells were infected as in (a) and AHA added 30 min prior to fixation at 8 hpi. AHA metabolic labelling was visualised using click‐it reaction (green) followed by staining for G3BP (magenta) and DNA (blue). Scale bars = 5 μm. (d) Cells from (c) were scored and quantified for the presence or absence of G3BP1 and active translation (AHA) within replication sites. Graph represents the mean + 1SD (*n* > 75). (e) HeLa cells were infected with WT WR at MOI 10 in the absence or presence of acrylamide. At 8 hpi, cells were fixed and processed for electron microscopy. Representative images of replication sites seen in the absence and presence of acrylamide (C = crescent, IV = intermediate virion, IVN = intermediate virion with nucleoid, D = dense region). All experiments were performed in triplicate

To determine if acrylamide was stimulating the formation of AVGs, HeLa cells were mock‐ infected or infected with WR mCh‐A5 in the absence or presence of acrylamide. At 8 hpi, cells were fixed, stained for nuclei and replication sites using Hoechst, and immunofluorescence performed for G3BP1 and eIF4G, two components of AVGs. In uninfected cells, irrespective of the presence of acrylamide, G3BP1 and eIF4G were diffusely distributed throughout cells with no evidence of SG formation (Figure 4a). A similar distribution was observed in untreated infected cells; G3BP1 and eIF4G staining was diffused throughout cells and largely excluded from viral replication sites (Figure 4a). However, in acrylamide‐treated infected cells both G3BP1 and eIF4G showed distinctive localisation to VACV replication sites, consistent with the formation of stress‐induced AVGs. That these structures were only formed in infected cells treated with acrylamide confirms that they are stress‐induced AVGs (Rozelle et al., 2014).

Acrylamide is an inducer of oxidative cell stress, which leads to phosphorylation of EIF2α and translational arrest (Komoike & Matsuoka, 2016, Komoike & Matsuoka, 2019). As our results indicated that VACV translation is inhibited by acrylamide, we assessed the effect of acrylamide treatment on eIF2α phosphorylation. For this, uninfected and VACV‐infected cells incubated in the absence or presence of acrylamide were harvested at 8 hpi and subjected to immunoblot analysis directed against eiF2α phosphorylation (Figure 4b). Despite the high background levels observed in HeLa cells, a 2.1 to 2.3‐fold increase in eIF2α phosphorylation was observed upon acrylamide treatment in both uninfected and infected cells (Figure 4b; right). This result is consistent with acrylamide‐mediated inhibition of VACV translation and the formation of stress‐induced AVGS.

As AVGs are known to form when VACV translation is blocked, we next looked at nascent protein synthesis. Again, cells were infected with WR mCh‐A5 in the absence or presence of acrylamide. To visualise active translation, the amino acid analogue Click‐iT L‐azidohomoalanine (AHA) was added to cells 30 min prior to fixation at 8 hpi. Cells were stained for nuclei and replication sites using Hoechst, and immunofluorescence performed for G3BP1. In untreated cells, active translation (AHA) was clearly seen within VACV replication sites while G3BP1 remained diffusely spread throughout the cell (Figure 4c). The converse was seen in the acrylamide‐treated cells, in which negligible levels of active translation were observed and G3BP1 strongly localised to VACV replication sites.

To quantify this phenotype, each cell was scored as containing clear G3BP1 puncta within replication sites or not (+G3BP/−G3BP) (Figure 4d). These two categories were then further divided into cells with evident AHA accumulation within the replication site and those without (+translation/−translation). When the percentage of cells within these four subcategories was determined, two clear cell populations emerged: in the untreated cells the majority of replication sites were translation (AHA) positive and G3BP1 (AVG) negative, whereas in acrylamide‐treated cells most replication sites were translation (AHA) negative and G3BP1 (AVG) positive (Figure 4d). These results are consistent with previous reports that G3BP1 localisation to replication sites correlates with a lack of viral gene translation (Rozelle et al., 2014).

### 2.5 Acrylamide treatment blocks infection prior to the initiation of VACV morphogenesis

As the acrylamide‐mediated block of VACV infection was not related to vimentin‐assisted formation of replication sites and virions, we wanted to visualise the impact of acrylamide on these viral structures. HeLa cells were infected with WT VACV in the absence or presence of acrylamide and processed for transmission electron microscopy at 8 hpi. In the untreated control sample, viral intermediates including crescents (C), immature virions (IVs) and immature virions with nucleoid (IVN) were observed (Figure 4e). In the presence of acrylamide however, early viral replication sites wrapped in endoplasmic reticulum (Punjabi & Traktman, 2005) were seen. While no discernible viral intermediates were present, the replication sites contained areas of increased density (D) often associated with early morphogenesis blocks (DeMasi & Traktman, 2000; Mercer & Traktman, 2005; Szajner, Weisberg, & Moss, 2004). These results confirm and extend our finding that acrylamide inhibits the assembly of nascent VACV virions through inhibition of late protein synthesis, a prerequisite of virion assembly.

## 3 DISCUSSION

The complex cytoplasmic lifecycle of VACV has been the subject of intense study for nearly 70 years (Moss, 2013). Despite this, the contribution of cellular factors to morphogenesis of poxvirus particles is scarcely defined (Condit et al., 2006). The intermediate filament vimentin was an exception, having been found in viral replication sites and virions, it was suggested to co‐ordinate the assembly of both (Manes et al., 2008; Resch et al., 2007; Risco et al., 2002). As vimentin is important for cytosolic organisation, maintenance of the microtuble network and cell integrity (Ivaska et al., 2007; Lowery et al., 2015), we wanted to better understand its role in VACV replication and assembly.

Following on from studies investigating the role of vimentin in virus infection, we employed acrylamide to collapse the vimentin filament network (Bhattacharya et al., 2007; Cordo & Candurra, 2003). While known to have multiple effects, in the absence of commercially available vimentin polymerisation inhibitors, acrylamide is a useful tool for studying the role of these intermediate filaments (Durham et al., 1983). We established that treatment of infected cells with acrylamide‐reduced VACV yields by >99% without impacting cell viability. Refining the stage of virus infection, we found partial inhibition of intermediate gene expression and genome replication culminating in a total of block late gene expression and subsequent virus assembly.

To verify that acrylamide‐mediated inhibition of VACV replication was due to disruption of the vimentin network, we generated a vimentin‐null HeLa cell line. We found that the absence of vimentin did not impact VACVs ability to form replication sites or to produce new infectious particles. We explored the possibility that the collapse of vimentin filaments around VACV replication sites, rather than its absence or general disruption, could be responsible for the block in virus assembly seen in the presence of acrylamide. However, treatment of VACV‐infected vimentin‐null cells with acrylamide still blocked infection. This finding diverges from other studies that used acrylamide to dissect the role of vimentin in virus infection (Bhattacharya et al., 2007; Cordo & Candurra, 2003; Fay & Pante, 2013; Issac et al., 2014; Miller & Hertel, 2009).

Despite our finding that vimentin removal has no impact upon VACV replication, it is found in replication sites and is packaged into VACV virions. Consistent with the role of this intermediate filament in cellular organelle positioning (Minin & Moldaver, 2008) perhaps vimentin plays a supportive, as opposed to essential, role in the VACV lifecycle. It will be of future interest to explore the organisation of viral replication sites or virion sub‐structures, in particular LBs, in the absence of vimentin.

Having uncoupled the effect of acrylamide on VACV infection from its ability to collapse the vimentin network, we turned our attention to other effects of acrylamide. A documented outcome of acrylamide exposure is the induction of oxidative and ER stress response pathways leading to the phosphorylation of eIF2α (Komoike & Matsuoka, 2016; Komoike & Matsuoka, 2019). The consequence of this is translational arrest, which can lead to the formation of cytosolic membraneless organelles termed SGs (Guzikowski et al., 2019). SGs have been shown to play a role in cellular‐antiviral response by inhibiting the accumulation of viral proteins upon oxidative stress (signalling via HRI kinase), ER stress (signalling via PERK) and upon sensing of dsRNA (signalling via PKR) (Farrell, Balkow, Hunt, Jackson, & Trachsel, 1977; Galabru & Hovanessian, 1987; Harding, Zhang, Bertolotti, Zeng, & Ron, 2000; McEwen et al., 2005; Onomoto, Yoneyama, Fung, Kato, & Fujita, 2014; Piotrowska et al., 2010). In turn, viruses have evolved mechanisms to manipulate SG formation and associated signalling pathways (McCormick & Khaperskyy, 2017).

AVGs are inhibitory cytoplasmic formations, which share similarities to SGs and form spontaneously at low levels during normal VACV infection, or at higher levels when cells are subject to stimuli such as oxidative stress or altered RNA helicase activity (Liem & Liu, 2016; Rozelle et al., 2014; Simpson‐Holley et al., 2011). To counteract AVG formation, VACV encodes the E3 protein that binds to and masks dsRNA thereby prevent host recognition and PKR activation (Chang, Watson, & Jacobs, 1992; Watson, Chang, & Jacobs, 1991). Given the block in post‐replicative gene expression, we investigated the possibility that acrylamide was inducing the formation of AVGs. Consistent with their formation, G3BP1 and eIF4G localised to nearly 100% of replication sites in acrylamide‐treatment infected cells. Consistent with stress‐induced AVG formation treatment of infected, but not infected cells, with acrylamide resulted in increased eIF2 phosphorylation and a lack of active translation within AVG‐containing replication sites.

**FIGURE 5:**
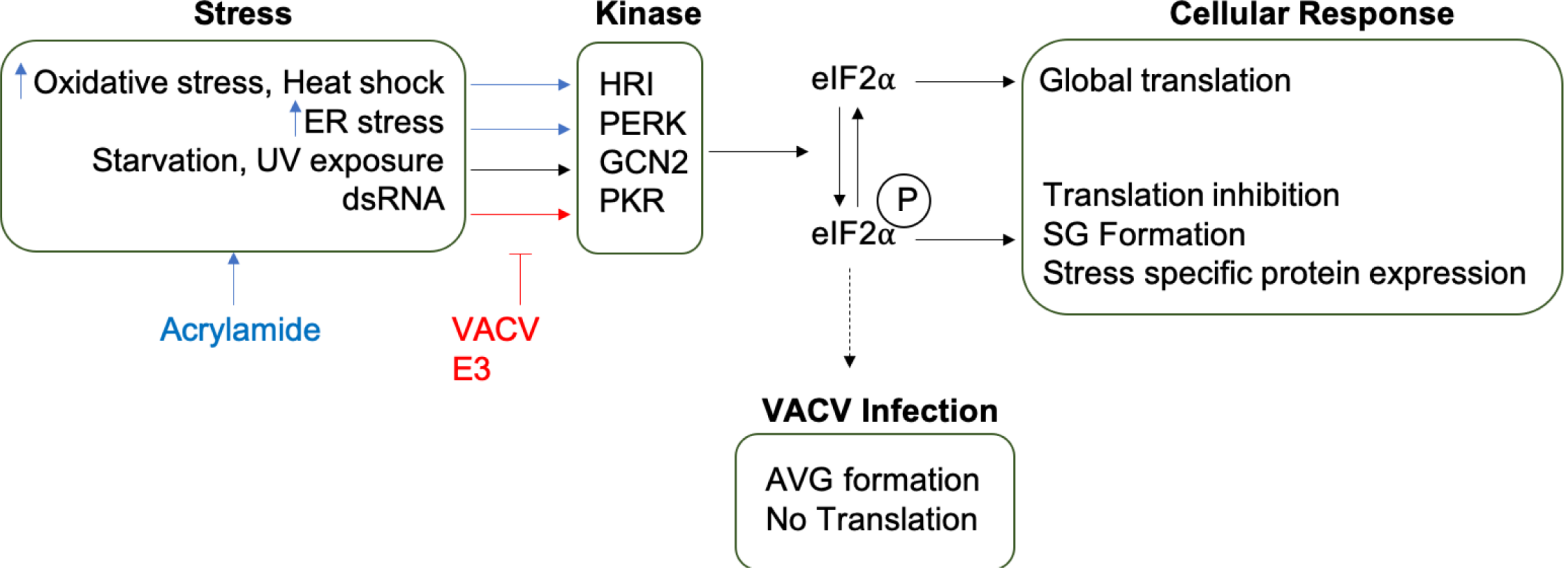
Mechanism of vaccinia virus (VACV) inhibition. We hypothesise that acrylamide activates non‐PKR‐ mediated cellular stress response pathway(s) which promote eIF2 phosphorylation‐mediated inhibition of VACV transcription/translation and the formation of antiviral granules

As illustrated in Figure 5, we propose that acrylamide activates non‐PKR mediated cellular stress response pathway(s) which promote eIF2 phosphorylation and subsequent formation of AVGs. This results in the slowing of viral genome replication and post‐replicative intermediate transcription/translation. VACV gene expression occurs in temporal waves with intermediate genes serving as late transcription factors. Thus, the inhibition of viral translation by AVGs has a knock‐on effect leading to a total block in VACV late gene transcription/translation culminating in the lack of late gene products required for assembly of nascent VACV virions. These results suggest, consistent with previous reports (Rozelle et al., 2014), that exogenous stimulation of AVG formation may be a useful strategy to promote cellular antiviral responses that bypass mechanisms used by VACV to manipulate host cell signalling.

## 4 EXPERIMENTAL PROCEDURES

### 4.1 Cell culture

BSC40 (African green monkey), HeLa and vimentin‐null HeLa cells were maintained at 37°C and 5% CO_2_ in complete Dulbecco's modified Eagle's medium (DMEM) containing 10% foetal bovine serum (FBS), 2 mM GlutaMAX and 1% penicillin–streptomycin. The vimentin‐null cell line was generated by transfection of a vimentin Double Nickase plasmid mix (Santa‐Cruz sc‐400035‐NIC). HeLa cells, at approximately 70% confluency, were transfected with 1 μg of the plasmid mix using Lipofectamine 2000 as per the manufacturer's instruction. Twenty‐four hours later, successful co‐transfection of the knockout plasmid (GFP) and the repair plasmid (RFP) was verified by visual inspection. Cells were then subjected to puromycin‐based selection. A puromycin concentration of 1 μg/ml was used as determined by a killing curve titration of puromycin on HeLa cells. Single puromycin‐resistant cells were monitored for colony formation. Single colonies, selected using glass cloning cylinders, were transferred to new plates and expanded. Once sufficient cell numbers were obtained, cell lines were assayed for vimentin expression as described.

### 4.2 Antibodies and reagents

The following antibodies were used for immunofluorescence; anti‐vimentin (Abcam ab20346, 1:250), anti‐vimentin (Sigma‐Aldrich V5255, 1:200), anti‐eIF4G1 (Abcam ab47649,1:400), anti‐G3BP (Abcam ab56574, 1:400), anti‐α‐tubulin (Cell signalling technology 3873S, 1:1,000), anti‐LaminB1 (Abcam ab133741, 1:200), anti‐β‐Actin (Sigma‐Aldrich A1978, 1:400), Alexa fluor secondary antibodies (Invitrogen, 1:500). For western blotting the following antibodies were used; anti‐A10 (Immune Technology IT‐012‐010M1, 1:2,000), anti‐A17 (1:1,000), anti‐F17 (1:2,500), anti‐vimentin (Sigma‐Aldrich V5255, 1:1,000), anti‐phospho‐eIF2α (Abcam ab131505, 1:1,000), anti‐α‐tubulin (Cell signalling technology 3873S, 1:5,000). Drugs were used at the following concentrations; 10 μM Cytosine arabinoside (AraC), 50 μM MG132, 3 μg/ml actinomycin D (ActD), 1 mM cycloheximide (CHX), 30 μM nocodazole, 4 mM acrylamide. When treating cells, acrylamide was added at 1 hpi, following the removal of inoculation media and unbound virions. For quantification, phospho‐eIF2α signals were determined by densitometry and normalised to the tubulin loading control. Untreated sample values were set at 1, and the average fold‐change in phospho‐eIF2α signal determined.

### 4.3 Viruses, VACV purification and infections

All viruses used in this study were derived from the VACV western reserve (WR) strain. In addition to wild‐type (WT WR), recombinant WR mCherry‐A5 (Schmidt et al., 2011), WR early EGFP (WR E‐GFP) and WR late EGFP (WR L‐GFP) viruses (Kilcher et al., 2014) were used as described previously. Viruses were purified by sedimentation through a sucrose cushion. Briefly, infected BSC40 cells were scraped into PBS and harvested at 300 g for 5 min. The pellet was then resuspended in 10 mM Tris, pH 9.0 and incubated on ice for 5 min. Cells were disrupted in a tight‐fitting Dounce homogeniser before centrifugation at 2,000*g*, 10 min, 4°C. The clarified supernatant was collected, and this centrifugation step repeated. The supernatant was then loaded over 36% sucrose in 20 mM Tris, pH 9.0 and then harvested at 38,000*g* 4°C for 80 min. The resulting pellet was resuspended in 200 μl 1 mM Tris and stored at −80°C, titres were determined by plaque assay as described below.

Infections were carried out at the specified MOIs in minimal volumes of serum free DMEM. VACV was thawed and virions resuspended with several rounds of sonication in a water bath. Virus dilutions were incubated on cell monolayers for 1 hr with agitation every 15 min. Cells were then fed with complete DMEM and incubated in conditions as indicated.

### 4.4 24‐hour yield and plaque assays

Confluent cells were infected with the appropriate viruses in 60 mm dishes at an MOI of 1 for 24 hr. Cells were then scraped into PBS and harvested at 700 g, 4°C for 5 min. The cell pellet was suspended in 100 μl 1 mM Tris pH 9.0 and subjected to three rounds of freeze thawing in liquid nitrogen. Plaque assays were performed by titrating harvested virus onto confluent monolayers of BSC40 cells in six well dishes. Cells were infected with 10‐fold dilutions of VACV and incubated for 48 hr before being stained with 0.1% crystal violet in 3.7% PFA.

### 4.5 Immunofluorescence

For immunofluorescence analysis, DMEM was removed and cells were fixed in 4% (vol/vol) paraformaldehyde in PBS and permeabilised in 0.2% (vol/vol) Triton X‐100 in PBS for 15 min each, with three PBS washes after each incubation. Alternatively, cells were fixed and permeabilised by incubating on ice with cold methanol for 10 min. Bovine serum albumin (BSA) at 1% (wt/vol) in PBS was used as a blocking agent and incubated with the cells for a minimum of 1 hr. Coverslips were incubated with primary antibodies diluted in blocking agent for 1 hr at indicated concentrations. Secondary antibodies were diluted in blocking agent and incubated with the cells for 1 hr. Coverslips were stained with Hoechst (1 μg/ml in PBS) for 15 min before mounting with Immu‐Mount (Thermo Scientific) and sealing with nail polish. Five washing steps were performed after each staining step and all incubation steps were carried out at room temperature, protected from light unless otherwise stated. Fluorescence microscopy was performed using a 100× oil immersion objective (NA 1.45) on a VT‐iSIM microscope (Visitech; Nikon Eclipse TI), using 405, 488, 561, 647 nm laser frequencies for excitation. Z‐stacks were generated from images taken at 0.20–0.25 μm intervals. Images were processed and analysed using NIS‐Elements AR software and Image J 2.0. All images were acquired at the same magnification and cropped for presentation purposes.

### 4.6 Visualisation of nascent protein synthesis

Metabolic labelling and visualisation were done using Click‐iT AHA (l‐azidohomoalaine) and the Click‐iT Cell Reaction Buffer Kit (Invitrogen), according to the manufacturer's instructions. Briefly, cells to be analysed were incubated with DMEM containing no glutamine, methionine or cystine for 1 hr prior to fixation. AHA was then added to cells at a concentration of 50 μM for an additional 30 min before fixation with 4% PFA and permeabilisation with 0.2% Triton for 15 min each. The click‐ it reaction cocktail was prepared containing an Alexa Fluor 488 Alkyne and incubated on coverslips for 30 min protected from the light. Coverslips were then washed and processed as per the immunofluorescence protocol above.

### 4.7 Flow cytometry analysis of VACV gene expression

HeLa cells were seeded into 24 well dishes, treated with appropriate drugs and infected at MOI 4 with either WR E‐GFP, or WR L‐GFP viruses for 6 and 8 hr, respectively. For analysis by flow cytometry, cells were washed with PBS and removed from the plate surface by trypsin treatment. Equal volumes of blocking buffer (5% FBS in PBS) and PFA (4% final concentration in PBS) were added to each well and incubated for 15 min. Samples were then transferred to 96 well plates ready for analysis by flow cytometry. All solutions were prewarmed to 37°C and all conditions were performed in triplicate. The Guava easyCyte HT instrument was used in conjunction with the Guava soft Incyte 3.1 software for analysis.

### 4.8 RT‐qPCR

For analysis of viral gene expression, HeLa cells were grown in 6 well dishes and infected in triplicate with WT WR at a MOI of 10 for either 2 hr (early), 4 hr (intermediate) or 8 hr (late). For CRISPR analysis, HeLa and vimentin‐null cells were grown to confluency in six well dishes before harvesting. The Qiagen RNeasy kit was used to extract RNA according to the manufacturer's instructions. RNA concentration was measured using the Nanodrop 2000 Spectrophotometer and 1 μg per sample was used as template for reverse transcription. cDNA was generated using SuperScript II reverse transcriptase (Thermo Fisher Scientific) and oligo(dT) primers. qPCR reactions were carried out using Mesa Blue qPCR MasterMix and appropriate primers on the BioRad CFX connect qPCR machine. Early samples were analysed with VACV J2R (5′‐TACGGAACGGGACTATGGAC‐3′ and 5′‐GTTTGCCATACGCTCACAGA‐3′) primers, intermediate with G8R (5′‐AATGTAGACTCGACGGATGAGTTA‐3′ and 5′‐TCGTCATTATCCATTACGATTCTAGTT‐3′) primers, late with F17R (5′‐ATTCTCATTTTGCATCTGCTC‐3′ and 5′‐AGCTACATTATCGCGATTAGC‐3′) primers and vimentin with (5′‐GCTCGTCACCTTCGTGAATA‐3′ and 5′‐CAGAGGGAGTGAATCCAGATTAG‐3′) primers. GAPDH primers were used on all samples as a housekeeping control. Fold change in mRNA levels was calculated using threshold cycle (*C*_*T*_) values normalised against the housekeeping control.

### 4.9 Quantification of viral DNA by qPCR

VACV genome replication was quantified by qPCR as previously described (Huttunen & Mercer, 2019). In short, cells were scraped into PBS and harvested at 400 g for 5 min. Genomic DNA was then extracted using the DNeasy Blood & Tissue Kit (Qiagen) as per manufacturer's instructions. A VACV genomic DNA dilution series of known concentration was used to create a standard curve. Samples were analysed by qPCR using the Mesa Blue qPCR MasterMix and C11R primer (5′‐AAACACACACTGAGAAACAGCATAAA‐3′, 5′‐ACTATCGGCGAATGATCTGATTA‐3′) BioRad CFX connect qPCR machine.

### 4.10 Electron microscopy

HeLa cells on coverslips were infected at MOI 10 with WT WR and treated as indicated. At 8hpi, the coverslips were fixed in EM‐grade 2% paraformaldehyde/2% glutaraldehyde (TAAB Laboratories Equipment, Ltd.) in 0.1 M sodium cacodylate, secondarily fixed for 1 hr in 1% osmium tetraoxide/1.5% potassium ferricyanide at 4°C and then treated with 1% tannic acid in 0.1 M sodium cacodylate for 45 min at room temperature. Samples were then dehydrated in sequentially increasing concentration of ethanol solutions, and embedded in Epon resin. Coverslips were inverted onto prepolymerised Epon stubs and polymerised by baking at 60°C overnight. The 70 nm thin sections were cut with a Diatome 45° diamond knife using an ultramicrotome (UC7; Leica). Sections were collected on 1 × 2 mm formvar‐coated slot grids and stained with Reynolds lead citrate. All samples were imaged using a transmission electron microscope (Tecnai T12; FEI) equipped with a charge‐coupled device camera (SIS Morada; Olympus).

### 4.11 Virus fractionations and western blotting

Purified WT WR virus was pelleted at 16,000 g, room temperature for 30 min and resuspended in fractionation buffer (1% NP‐40, 50 mM DTT in 1 mM Tris pH 9.0). After incubation at 37°C for 30 min, samples were centrifuged at 16,000*g*, 4°C for 30 min. Pellets (core and LB fraction) were resuspended in 10 mM Tris pH 9.0 and supernatants (membrane fraction) transferred to a fresh tube. To remove LBs from cores, the combined fraction was resuspended in varying concentrations of trypsin (from 0.125 to 1 μg/ml made up in 10 mM Tris pH 9.0) and incubated at 37°C for 15 min. Trypsin inhibitor was then added followed by a 15 min incubation at 25°C. Samples were centrifuged at 15,000 rpm, 4°C for 30 min, the supernatant was transferred to a fresh tube and the core containing pellets resuspended in 10 mM Tris pH 9.0. For analysis, samples were boiled with Laemmli buffer and separated on SDS‐PAGE gels prior to transfer of proteins onto nitrocellulose membranes. Proteins were visualised using indicated antibodies on the Li‐Cor Odyssey 3.0 imaging system.

### 4.12 Cytotoxicity assay

Cytotoxicity was measured using the Pierce LDH Cytotoxicity Assay Kit (Thermo Scientific), following the manufacturer's instructions. Briefly, staurosporine 10 mM (positive control), DMSO 1% (negative control), acrylamide 4 mM or nocodazole 30 μM was added to 70–80% confluent HeLa cells in a 96‐well plate. After 24 hr at 37°C, 50 μl of sample media was mixed with 50 μl of Reaction Mix in a new plate and incubated at room temperature for 30 min, protected from light. Absorbance at 490 and 680 nm was measured using the FLUOstar Omega (BMG Labtech) after addition of 50 μl of Stop Reaction. LDH activity was calculated by subtracting the 680 nm absorbance value from the 490 nm absorbance value and readings normalised to staurosporine.

## CONFLICT OF INTEREST

The authors declare no potential conflict of interest.

## AUTHOR CONTRIBUTION

Jennifer J. Wood and Jason Mercer designed the study. Jennifer J. Wood Wood performed and analysed experiments. Ian J. White prepared and imaged EM samples and Jerzy R. Samolej performed toxicity assays. Jennifer J. Wood and Jason Mercer wrote the manuscript with input from Ian J. White and Jerzy R. Samolej.

## Data Availability

The data that support the findings of this study are available from the corresponding author upon reasonable request.
